# The Characteristics and Two-Year Neurodevelopmental Outcomes of Home Oxygen Therapy among Preterm Infants with Bronchopulmonary Dysplasia: A Retrospective Study in a Medical Center in Taiwan

**DOI:** 10.3390/biomedicines12071564

**Published:** 2024-07-15

**Authors:** Han-Pi Chang, En-Pei Lee, Ming-Chou Chiang

**Affiliations:** 1 Division of Pediatric Emergency, Department of Pediatrics, Linkou Chang Gung Memorial Hospital, Taoyuan 333, Taiwan; blueahbi7903l@cgmh.org.com; 2 College of Medicine, Chang Gung University, Taoyuan 333, Taiwan; lnp1511@cgmh.org.tw; 3 Division of Pediatric Critical Care Medicine, Department of Pediatrics, Linkou Chang Gung Memorial Hospital, Taoyuan 333, Taiwan; 4 Division of Neonatology, Department of Pediatrics, Linkou Chang Gung Memorial Hospital, Taoyuan 333, Taiwan; 5 Division of Respiratory Therapy, Linkou Chang Gung Memorial Hospital, Taoyuan 333, Taiwan

**Keywords:** bronchopulmonary dysplasia, home oxygen therapy, neurodevelopmental outcome, preterm infants, very low-birth-weight infants

## Abstract

Home oxygen therapy (HOT) is frequently used as a therapeutic strategy for children experiencing chronic oxygen dependency associated with bronchopulmonary dysplasia (BPD). Recent studies have highlighted substantial variations in the characteristics and outcomes of infants requiring oxygen, primarily due to the absence of a consensus on the management of HOT in infants with BPD. We conducted this retrospective study and reviewed the medical records of extremely and very preterm infants who were diagnosed with BPD in a tertiary center in northern Taiwan from January 2020 to September 2021. Their neurodevelopmental outcomes were evaluated at 18 to 24 months of corrected age. A total of 134 patients diagnosed with BPD were divided into a HOT group (*n* = 39) and a room air group (*n* = 95). The children in the HOT group had a higher incidence of hemodynamic significant patent ductus arteriosus (PDA) (*p* = 0.005) and PDA ligation (*p* = 0.004), high-frequency oscillatory ventilation (*p* < 0.001), nitrogen oxide inhalation (*p* < 0.001), pulmonary hypertension (*p* = 0.01), and longer invasive ventilation (*p* < 0.001), as well as longer hospitalization (*p* < 0.001). A multivariate logistic regression model demonstrated that prolonged invasive ventilation (OR = 1.032, 95% CI 0.984–1.020, *p* = 0.001) was correlated with oxygen dependency in children. Infants with BPD born at advanced gestational age (OR = 0.760, 95%CI 0.582–0.992, *p* = 0.044) had a decreasing risk of requiring HOT. The children in the HOT group had a higher incidence of emergency room visits (*p* < 0.001) and re-hospitalization (*p* = 0.007) within one year of corrected age. The neurodevelopmental outcomes revealed the HOT group had an increasing portion of moderate to severe cognitive delay (18.2% vs. 3.7%, *p* = 0.009) and moderate to severe language delay (24.2% vs. 6.1%, *p* = 0.006) at 18 to 24 months of corrected age. In conclusion, infants with BPD necessitating HOT required prolonged invasive ventilation during hospitalization and exhibited a greater prevalence of unfavorable neurodevelopmental outcomes at 18 to 24 months of corrected age as well.

## 1. Introduction

Bronchopulmonary dysplasia (BPD) stands as a prevalent complication in extremely preterm neonates, with rates from 18 to 82% in Asia [1]. In Taiwan, the incidence of BPD is on the rise, primarily due to advancements in the survival rates of very low-birth-weight infants [2]. Consequently, there has been a notable increase in the number of extremely preterm infants who need home oxygen support upon discharge [3].

Home oxygen therapy (HOT) is commonly advised for infants diagnosed with BPD or chronic lung disease who demonstrate stability while using low-flow oxygen nasal cannula [4,5]. The implementation of HOT enables children’s discharge and for them to be cared for at home. The positive effects of HOT not only improved the neurodevelopment of infants and strengthened the relationship between parents and children but also reduced costs compared to extended hospitalization [6]. However, the clinical practice in HOT in BPD patients varies significantly among hospitals, both nationally and internationally [7,8]. The diversity in the management of HOT for infants with BPD is due to the lack of well-established knowledge regarding the risks and benefits associated with the prolonged used of supplemental oxygen in the newborn period [6,9]. The variation in applying HOT results in the outcomes of BPD patients who required supplemental oxygen remaining uncertain [10,11,12]. Currently, information for evaluating the characteristics and long-term outcomes in patients with BPD who required supplemental oxygen at home is lacking in Taiwan.

Therefore, our study aimed to elucidate the factors, clinical features, and outcomes associated with preterm infants diagnosed with BPD who required HOT. By identifying these variables, we hope to enhance our understanding, thereby contributing to the development of targeted interventions, informed policy making, and comprehensive evaluation of long-term outcomes for pediatric individuals undergoing HOT in forthcoming research and clinical practice.

Through comprehensive analysis and characterization of these factors, we hope to contribute insights into optimizing the care and the management of preterm infants with BPD, ultimately improving their overall health outcomes and quality of life. Additionally, our findings may provide information for future guidelines and protocols surrounding the use of HOT in this vulnerable population, ensuring that infants receive the appropriate care to support their growth and development.

## 2. Materials and Methods

### 2.1. Study Design and Patients

This was a retrospective, single-center study conducted in Linkou Chang Gung Memorial Hospital during the period between January 2020 and September 2021. We collected clinical data including preterm infants whose birth weight (BW) was less than 1500 g or gestational age (GA) was less than 32 weeks. Their demographic data and clinical characteristics were obtained through electronic medical records. The diagnosis and grading of BPD were according to the 2018 National Institute of Child Health and Human Development consensus [13]. Infants diagnosed with BPD were divided into a room air group and a home oxygen therapy group based on their requirement for oxygen supplementation at discharge. Patients with congenital heart disease, anomalies of the airways, and chromosomal abnormalities were excluded.

### 2.2. Clinical Variables

Early-onset sepsis (EOS) was characterized by positive blood cultures for bacteria within 1 week of life, whereas late-onset sepsis (LOS) was defined by positive blood cultures for bacteria occurring after the first week of life [14]. Hemodynamically significant patent ductus arteriosus (hsPDA) was identified according to one of the following criteria [15]: (1) a PDA diameter ≥1.5 mm, (2) unrestrictive pulsatile flow through the ductus, (3) a left atrial-to-aortic root ratio ≥1.5, or the absence of end-diastolic flow in the descending aorta. The confirmation of pulmonary hypertension involved the performance of echocardiography at a minimum of 28 days of age (beyond 36 weeks of corrected gestational age or prior to discharge). Pediatric cardiologists conducted thorough screenings of all the echocardiographic assessments. Shunt directionality via an atrial septal defect, patent foramen ovale, or PDA was assessed as either left-to-right, right-to-left, or bidirectional. The pediatric cardiologists documented findings such as septal flattening, right ventricular (RV) hypertrophy, and dilatation. RV systolic pressure was estimated based on the tricuspid regurgitant jet velocity. The diagnostic criteria for pulmonary hypertension comprised (1) an RV systolic pressure exceeding 40 mm Hg; (2) the presence of bidirectional or right-to-left cardiac shunting; and (3) the identification of interventricular septal flattening, RV hypertrophy, or dilatation in the absence of residual shunting, including atrial septal defects, ventricular septal defects, or PDA [16].

### 2.3. Outcomes

The clinical characteristics and clinical outcomes included re-hospitalization, admission to the intensive care unit (ICU), and visits to the emergency room (ER), which were evaluated when they reached 12 months of corrected age. We assessed the infants’ neurodevelopmental outcomes by using the Bayley Scales of Infant and Toddler Development, third edition (Bayley-III, 2006) [17], at the age of 18 to 24 months of corrected age. The Bayley-III is widely recognized as a primary tool for evaluating infant development and diagnosing developmental delays in early childhood. There were 5 domains in the Bayley-III: cognition, motor, language, adaptive, and social/emotional development. The classifications of development delay were normal, within 1 SD of the mean (≥85); mild, −1 SD to −2 SD (≥70 and <85); moderate, −2 SD to 3 SD (≥55 and <70); and severe delay, less than −3 SD (<55).

### 2.4. Statistical Analysis

All statistical analyses were performed using the Stata software package, version 14.0 (StataCorp). Categorical variables were analyzed using the chi-square test or Fisher’s exact test, while the Mann–Whitney U test was utilized for continuous variables. To explore the factors associated with HOT, a multivariate logistic regression model was constructed. The model encompassed variables such as GA, BW, PDA ligation, days of invasive ventilation, and duration of hospital stays. Statistical significance was defined as a *p* value < 0.05.

## 3. Results

In our study, we recruited a total of 134 patients diagnosed with BPD. These patients were categorized into two groups: the HOT group (*n* = 39) and the room air group (*n* = 95). Our analysis revealed no statistically significant difference in terms of the maternal characteristics between the room air and HOT groups (Table 1).

The mean BW of infants with BPD was 997 ± 284 g, and their mean GA was 28 ± 2 weeks. Comparing the HOT group to the room air group, significant differences were noted in both BW (842 ± 283 vs. 1062 ± 274, *p* < 0.001) and GA (26 ± 2 vs. 29 ± 2, *p* < 0.001). Children in the HOT group exhibited a higher incidence of hsPDA (*p* = 0.005) and PDA ligation (*p* = 0.004) and greater utilization of high-frequency oscillatory ventilation (*p* < 0.001) and inhaled nitrogen oxide (*p* < 0.001). Additionally, they experienced prolonged durations of invasive ventilation (*p* < 0.001), a higher prevalence of pulmonary hypertension (*p* = 0.01), and longer hospitalization periods (*p* < 0.001) (Table 2).

In this study, we assessed the re-admission rate, ICU admission rate, and ER visit rate within 12 months of corrected age. We observed a notable increase in the rate of re-admission (*p* = 0.007) and ER visits (*p* < 0.001) among the patients in the HOT group. A total of 8 patients re-visited the ER because of cyanosis, while 14 patients were diagnosed with acute bronchiolitis or pneumonia during these visits. Our investigation regarding the neurodevelopmental outcomes revealed significant differences between the HOT group and the room air group. The HOT group had an increasing portion of moderate to severe cognitive delay (18.2% vs. 3.7%, *p* = 0.009) and moderate to severe language delay (24.2% vs. 6.1%, *p* = 0.006) at 18 to 24 months of corrected age (Table 3).

Upon conducting the multivariable analysis, it was found that prolonged invasive ventilation (OR = 1.032, 95% CI 0.984–1.020, *p* = 0.001) was associated with oxygen dependency in children. Infants with BPD and born at advanced gestational age showed a decreasing propensity to require HOT (OR = 0.760, 95% CI 0.582–0.992, *p* = 0.044) (Table 4).

## 4. Discussion

Our cohort analysis illustrated the clinical characteristics and outcomes in preterm infants with BPD and receiving home oxygen therapy in a medical center in northern Taiwan, which were similar to the findings from previous studies [18]. Notably, prolonged reliance on invasive ventilation was a predominant feature among these infants. Dassios et al. showed that infants requiring mechanical ventilation for extended periods, often exceeding 10 days, are at an increasing risk of requiring oxygen therapy upon discharge [19]. Prolonged exposure to invasive ventilation can trigger lung inflammatory responses and tissue damage, ultimately necessitating chronic oxygen supplementation in affected infants [20]. Thus, patients receiving longer invasive ventilation during hospitalization should be closely monitored after discharge when receiving home oxygen therapy.

A higher incidence of re-hospitalization within 12 months of corrected age among infants receiving HOT was shown in the current study. Acute bronchiolitis and pneumonia were common causes for re-hospitalization in our cohort, aligning with previous research demonstrating a correlation between prolonged oxygen use and increased susceptibility to respiratory illness [21,22]. A comprehensive study involving 1039 infants revealed infants with extended oxygen dependency were at higher risk of re-hospitalization for recurrent respiratory illness, escalating the need for respiratory medications and support [11]. Hence, we recommend that infants on home oxygen therapy should be monitored closely, particularly for respiratory infections, which may result in increased rates of re-admission and burdens of medical costs.

Our study also showed that the infants in the HOT group exhibited lower cognitive and language scores. Our findings differed from those reported by DeMauro et al., who observed no significant difference in the neurodevelopmental outcomes between their two groups [11] (Table 5). This difference in outcomes may stem from the diverse strategies employed for home oxygen therapy among hospitals. The variability in clinical practice could account for the discrepancies between our findings and those reported by Dr. DeMauro. Strategies for weaning off home oxygen are primarily guided by expert opinions and clinical experience. Research on the optimal oxygen saturation during weaning oxygen is limited, which may result in some children not receiving sufficient oxygen to meet their developmental needs. Inappropriate weaning from oxygen may cause chronic hypoxia in children, potentially affecting their neurological outcomes [23]. Moreover, the HOT group had longer hospital stays compared to the room air group in our cohort, whereas Dr. DeMauro’s study found no significant difference between the two groups. The variation in the length of the hospital stays may have also impacted the results of the study. A longer hospital stay has been proven to adversely impact on developmental outcomes [24]. Our study concurred with their findings regarding the duration of hospitalization and the utilization of invasive mechanical ventilation within the HOT cohort. Prolonged respiratory support beyond 60 days, whether invasive or non-invasive, demonstrated an elevated association with an increased risk of unfavorable neurodevelopmental outcomes [25]. Extended hospitalization and prolonged respiratory support were identified as contributing factors to adverse neurodevelopmental outcomes among patients with BPD as well [26]. Thus, we speculated that the poor neurodevelopmental outcomes observed in the HOT group may be attributable to the absence of evidence-based protocols for weaning home oxygen, prolonged respiratory support, and extended hospitalization.

Infants with BPD and receiving home oxygen therapy are at a higher risk of having events of deoxygenation. Chronic, intermittent, or prolonged deoxygenation, if it occurs, is assumed to be associated with adverse neurodevelopmental outcomes among infants receiving HOT. Studies have shown the association between chronic hypoxemia and adverse neurodevelopmental sequalae [27,28]. Infants requiring oxygen therapy upon discharge are at increased risk of developing pulmonary hypertension [29]. Pulmonary hypertension can result from prolonged exposure to hypoxemia and respiratory distress, leading to pulmonary vascular remodeling and increased pulmonary vascular resistance [30,31,32]. These hemodynamic alterations may compromise cerebral perfusion and oxygen delivery, thereby predisposing infants to neurodevelopmental impairments [33,34]. The pathophysiological mechanisms regarding pulmonary hypertension, such as vascular remodeling, inflammation, and oxidative stress, may exacerbate the neuroinflammatory process and disrupt neuronal integrity [35]. The children in the HOT group had a higher incidence of having pulmonary hypertension. However, we did not find direct evidence regarding the effects of pulmonary hypertension on the development of adverse neurodevelopmental outcomes. The mechanisms linking pulmonary hypertension and neurodevelopmental outcomes remain incompletely understood. More studies are needed to clarify the relationship between prolonged deoxygenation, pulmonary hypertension, and neurodevelopmental outcomes.

It is assumed that the inability to achieve a better neurodevelopmental outcome in patients with HOT might be due to the lack of a standardized protocol for gradually discontinuing oxygen support. To date, there are no universally acknowledge guidelines for weaning pediatric patients off oxygen undergoing HOT after discharge [9]. A survey among pediatric pulmonologists revealed that only 8% adhered to a standardized protocol for weaning off oxygen, with a diverse range of criteria employed for discontinuation [36]. Many infants were identified whose caregivers did not adhere to medical guidance when weaning them off oxygen [37]. Discontinuing oxygen therapy without proper supervision is worrisome, as the evidence indicates that insufficient oxygenation may compromise neurodevelopment and somatic growth [38]. Evidence on the optimal SpO2 levels and the rate of weaning off home oxygen to achieve optimal growth and development in children with chronic lung disease is limited [6]. The negative impacts of chronic or intermittent hypoxia when weaning off oxygen on development outcomes have been proven [23]. The evaluation of a sufficient oxygen supply for developmental and growth needs during weaning off oxygen should be established. Additionally, infants requiring prolonged oxygen therapy tend to have extended hospitalization, more respiratory support, and poor weight gain compared with infants successfully weaned off oxygen within 12 months [12]. Prolonged NICU stays and chronic pulmonary insufficiency have been demonstrated to negatively affect early developmental outcomes in children [39,40]. Children requiring a longer time to wean off oxygen exhibit poor pulmonary function, which may adversely impact their neurodevelopmental outcomes. Therefore, we recommended that patients requiring extended durations to wean off oxygen receive more comprehensive neurodevelopmental assessments and early interventions to gain better outcomes. We contend that the safe and comprehensive administration of home oxygen therapy, combined with closely monitoring for adverse effects in infants, will positively influence their growth and development. The guidelines should include the rate of weaning off oxygen, monitoring adverse effects, and assessment of growth and development. Implementing evidence-based guidelines for weaning off home oxygen could enhance the quality of care for and the neurodevelopmental outcomes in infants with BPD.

There were several limitations to the current study. First, the major limitation of this study is the relatively small sample size of our patient cohort, coupled with the retrospective design and its conduct solely at a single medical center. Such constraints inherently pose risks of both missing data and information bias. The limited sample size may not fully capture the breadth of the variability in the clinical presentations and outcomes among preterm infants, thus potentially limiting the generalizability of our findings to broader populations. Second, it was challenging to interpret the data and analyze the impacts on clinical outcomes with the variability of the duration of HOT among the study cohort. Third, there was no optimal target for pulse oximetry in infants with BPD and receiving HOT. While pulse oximetry serves as a valuable tool for monitoring oxygen saturation levels in infants receiving home oxygen therapy, the absence of standardized guidelines or consensus regarding target oxygen therapy ranges complicates clinical decision-making. This uncertainty may lead to variability in clinical practice and outcomes.

## 5. Conclusions

Prolonged use of invasive ventilation was associated with extended reliance on oxygen in infants with BPD. Compared with the room air group, infants requiring HOT demonstrated a higher proportion of unfavorable neurodevelopmental outcomes at 18 to 24 months of corrected age. The establishment of local HOT guidelines, aiming at safely weaning off oxygen support, is important and may have the potential to improve the neurodevelopmental outcomes in patients with BPD receiving HOT.

## Figures and Tables

**Table 1:** Maternal characteristics of infants with and without home oxygen therapy (HOT).

	Total (*n* = 134)	Room Air (*n* = 95)	HOT (*n* = 39)	*p* Value
Maternal Characteristics				
Cesearean section, *n* (%)	95 (71)	70 (74)	25 (64)	0.267
GDM, *n* (%)	12 (9)	11 (12)	1 (3)	0.097
Oligohydraminos, *n* (%)	4 (3)	4 (4)	0	0.193
Polyhydraminos, *n* (%)	5 (4)	4 (4)	1 (3)	0.648
Pre-eclampsia, *n* (%)	22 (16)	19 (20)	3 (8)	0.081
Chorioamnionitis, *n* (%)	28 (21)	19 (20)	9 (23)	0.691
PPROM, *n* (%)	66 (49)	45 (47)	21 (54)	0.496

GDM = gestational diabetes mellitus, PPROM = preterm premature rupture of membranes.

**Table 2:** Neonatal characteristics of infants with and without home oxygen therapy (HOT).

	Total (*n* = 134)	Room Air (*n* = 95)	HOT (*n* = 39)	*p* Value
Neonatal Characteristics				
Male, *n* (%)	74 (55)	52 (55)	22 (56)	0.86
GA (week), mean ± SD	28 ± 2	29 ± 2	26 ± 2	<0.001 *
BW (gram), mean ± SD	997 ± 284	1062 ± 274	842 ± 283	<0.001 *
SGA, *n* (%)	18 (13)	14 (15)	4 (10)	0.490
EOS, *n* (%)	11 (8)	5 (5)	6 (15)	0.053
LOS, *n* (%)	43 (32)	26 (27)	17 (44)	0.068
hsPDA, *n* (%)	62 (46)	37 (39)	25 (64)	0.005 *
PDA ligation, *n* (%)	41 (31)	22 (23)	19 (49)	0.004 *
HFOV, *n* (%)	50 (37)	26 (27)	24 (62)	<0.001 *
iNO, *n* (%)	16 (12)	4 (4)	12 (31)	<0.001 *
NEC, *n* (%)	21 (16)	16 (17)	5 (13)	0.561
IVH, *n* (%)	32 (24)	20 (21)	12 (31)	0.231
ROP, *n* (%)	54 (40)	29 (31)	25 (64)	<0.001 *
Pulmonary hypertension, *n* (%)	9 (7)	3 (3)	6 (15)	0.01 *
Duration of invasive ventilation (day), mean ± SD	35 ± 32	24 ± 26	60 ± 32	<0.001 *
Duration of oxygen support at NICU (day), mean ± SD	87 ± 42	73 ± 30	123 ± 46	<0.001 *
**Clinical outcome**				
Hospitalization (day), mean ± SD	133 ± 40	87 ± 30	125 ± 47	<0.001 *
Re-hospitalization within 1 year old, *n* (%)	14 (10.4)	5 (5.3)	9 (23.1)	0.007 *
Re-hospitalized for cyanosis, *n* (%)	8 (6)	1 (1.1)	7 (17.9)	<0.001 *
Re-hospitalized for respiratory disease, *n* (%)	14 (10.4)	6 (6.3)	8 (20.5)	0.013 *
ICU admission within 1 year old, *n* (%)	5 (3.7)	2 (2.1)	3 (7.7)	0.116
ER visit within 1 year old, *n* (%)	20 (14.9)	8 (8.4)	12 (30.8)	<0.001 *

GA = gestational age, BW = birth weight, SGA = small for gestational age, EOS = early-onset sepsis, LOS = late-onset sepsis, hsPDA = hemodynamic significant patent ductus arteriosus, PDA ligation = patent ductus arteriosus ligation, HFOV = high-frequency oscillatory ventilation, iNO = inhaled nitric oxide, NEC = necrotizing enterocolitis, IVH = intraventricular hemorrhage, ROP = retinopathy of prematurity, ICU = intensive care unit, ER = emergency room, * *p* value < 0.05.

**Table 3:** Bayley III performance score in infants with and without home oxygen therapy (HOT) at 18 to 24 months of corrected age.

	Room Air (*n* = 81)	HOT (*n* = 33)	*p* Value
**Cognitive score**			
Mild delay ≥ 70 and <85, *n* (%)	16/81 (19.8)	7/33 (21.2)	0.86
Moderate to severe delay < 70, *n* (%)	3/81 (3.7)	6/33 (18.2)	0.009 *
**Language score**			
Mild delay ≥ 70 and <85, *n* (%)	32/81 (39.5)	11/33 (33.3)	0.537
Moderate to severe delay < 70, *n* (%)	5/81 (6.1)	8/33 (24.2)	0.006 *
**Motor score**			
Mild delay ≥ 70 and <85, *n* (%)	14/81 (17.3)	10/33 (30.3)	0.122
Moderate to severe delay < 70, *n* (%)	4/81 (4.9)	4/33 (12.1)	0.173

* *p* value < 0.05.

**Table 4:** Risk factors for home oxygen therapy by multivariate logistic analysis.

Clinical Characteristics	Odds Ratio	95%CI	*p* Value
Gestational age	0.760	0.582–0.992	0.044 *
Birth weight	0.723	0.997–1.002	0.723
PDA ligation	0.764	0.267–2.189	0.616
Days of invasive ventilation	1.032	1.012–1.052	0.001 *
Duration of hospital stays	1.002	0.984–1.020	0.806

PDA = patent ductus arteriosus, * *p* value < 0.05.

**Table 5:** Comparison of outcomes in BPD infants with home oxygen therapy (HOT) and room air.

Bayley III Performance Score	Our Cohort			MSCE Study (2019) #		
	Room Air	HOT	*p* Value	Room Air	HOT	*p* Value
**Cognitive score**						
Moderate to severe delay < 70, *n* (%)	3/81 (3.7)	6/33 (18.2)	0.009 *	112/992 (11)	122/973 (13)	0.08
**Language score**						
Moderate to severe delay < 70, *n* (%)	5/81 (6.1)	8/33 (24.2)	0.006 *	212/960 (22)	210/973 (22)	0.48
**Motor score**						
Moderate to severe delay < 70, *n* (%)	4/81 (4.9)	4/33 (12.1)	0.173	107/764 (14)	107/74 (16)	0.61
**Characteristics**						
Duration of invasive ventilation (day), mean ± SD	24 ± 26	60 ± 32	<0.001 *	63 ± 25	61 ± 23	
Hospitalization (day), mean ± SD	87 ± 30	125 ± 47	<0.001 *	124 ± 34	120 ± 40	

# Reference [11]: DeMauro, S. B.; Jensen, E. A.; Bann, C. M.; Bell, E. F.; Hibbs, A. M.; Hintz, S. R.; Lorch, S. A. (2019). Home oxygen and 2-year outcomes of preterm infants with bronchopulmonary dysplasia. *Pediatrics*, *143*(5). * *p* value < 0.05.

## Data Availability

The data used to support the findings of this study are available from the corresponding author upon request.
